# An Effective Model for Screening Obstructive Sleep Apnea: A Large-Scale Diagnostic Study

**DOI:** 10.1371/journal.pone.0080704

**Published:** 2013-12-02

**Authors:** Jianyin Zou, Jian Guan, Hongliang Yi, Lili Meng, Yuanping Xiong, Xulan Tang, Kaiming Su, Shankai Yin

**Affiliations:** Department of Otolaryngology, The Affiliated Sixth People's Hospital, Otolaryngology Institute of Shanghai Jiao Tong University, Shanghai, China; Hospital General Dr. Manuel Gea González, Mexico

## Abstract

**Background:**

Obstructive sleep apnea (OSA) causes high morbidity and mortality and is independently associated with an increased likelihood of multiple complications. The diagnosis of OSA is presently time-consuming, labor-intensive and inaccessible.

**Aim:**

This study sought to develop a simple and efficient model for identifying OSA in Chinese adult population.

**Methods:**

In this study, the efficiency of Epworth Sleepiness Scale (ESS) and a new established prediction model for screening OSA were evaluated in the test cohort (2,032 participants) and confirmed in an independent validation cohort (784 participants).

**Results:**

In the test cohort, a high specificity (82.77%, 95% confidence interval [CI], 77.36–87.35) and a moderate sensitivity (61.65%, 95% CI, 59.35–63.91) were obtained at the threshold of nine for the ESS alone. Notably, sex-stratified analysis revealed different optimum cut-off points: nine for males and six for females. The new generated screening model, including age, waist circumference, ESS score, and minimum oxygen saturation (SaO_2_) as independent variables, revealed a higher sensitivity (89.13%, 95% CI, 87.60–90.53) and specificity (90.34%, 95% CI, 85.85–93.77) at the best cut-off point. Through receiver operating characteristics curve analysis, the area under the receiver operating characteristics curve of the model was found significantly larger than that of the ESS alone (0.955 vs. 0.774, *P*<0.0001). All these results were confirmed in the validation cohort.

**Conclusions:**

A practical screening model comprising minimum SaO_2_ and other parameters could efficiently identify undiagnosed OSA from the high-risk patients. Additionally, a sex-specific difference should be considered if the ESS alone is used.

## Introduction

Obstructive sleep apnea (OSA) is insidious, and patients are often unaware that OSA is associated with high morbidity [1]. Despite improved diagnostic procedures and an increasing awareness of the health consequences, a high rate of missed diagnosis of OSA persists. Simpson et al. [2] found that the prevalence of undiagnosed moderate-severe OSA in a general population sample from Western Australia was up to 9%. An epidemiological survey of OSA in Shanghai, China, showed that the prevalence of OSA was 3.62%, but only 0.54% had been treated; more than 85% of OSA was undiagnosed [3]. Evidence from methodologically strong cohort studies indicates that undiagnosed OSA is independently associated with the increased likelihood of hypertension, cardiovascular diseases, stroke, daytime sleepiness, motor vehicle accidents, and diminished quality of life [4]. If OSA persists, some symptoms and sequelae are irreversible [5]. Therefore, screening and early diagnosis of OSA is important to alleviate the major health-related consequences and developmental complications of the syndrome.

Although commonly used as the gold standard [6], [7] for diagnosis of OSA, nocturnal laboratory-based polysomnography (PSG) is considered time-consuming, labor-intensive, and costly. Therefore, many researchers have attempted to develop a simple and effective tool to screen patients with OSA.

The Epworth Sleepiness Scale (ESS), designed as a subjective method of estimating excessive daytime sleepiness (EDS), has been widely studied for the screening of OSA [8]. However, studies have reported conflicting conclusions regarding the efficiency of the ESS for screening OSA [9], [10].

Establishing a screening model that includes several parameters is another field of research for screening OSA. Many studies [11], [12] have revealed a variety of predictive parameters, such as snoring, witnessed apnea, hypertension, and neck circumference (NC), that were included in such models. However, these models were found to be insufficient due to a variety of limitations. Takegami *et al.* [12] reported a screening model with a high sensitivity (0.93) but a low specificity (0.66). Chai-Coetzer *et al.* [13] developed a two-stage model with a high diagnostic accuracy, however, that study focused on the identification of patients with moderate-to-severe OSA and did not evaluate the ability of the model to identify populations with mild OSA and snoring. Several parameters derived from nocturnal oximetry have also been developed to screen OSA. The frequently used parameters include oxygen desaturation index [13], and the cumulative time spent below saturation of 90% [14], but neither of them is widely popularized. Additionally, the value of minimum oxygen saturation (SaO_2_) was often ignored in predicting OSA, even it was strongly correlated with treatment response of OSA [15], [16]. According to these incidences, we presumed minimum SaO_2_ may be effective in predicting OSA.

Given the significant morbidity and high ratio of missed diagnosis associated with OSA, establishing a method for OSA screening is important. In particular, early diagnosis could reduce the incidence of serious OSA-related complications. Furthermore, investigation of factors predictive of OSA may facilitate the development of treatment and prevention strategies [17]. Therefore, we designed a large-scale validation study to evaluate the efficiency of the ESS and a new, more efficient screening model for the identification of patients suspected with OSA in a Chinese adult population.

## Methods

### Study design and population

The test cohort included unrelated consecutive subjects suspected as having OSA who were admitted to the Sleep Center of the Affiliated Sixth People's Hospital, Shanghai Jiao Tong University from January 2007–July 2011. Another group of unrelated consecutive participants recruited from August 2011–July 2012 were included in the validation cohort. The Institutional Ethics Committee of the Hospital of Shanghai Jiao Tong University approved the study. Informed consent was obtained from all participants. All participants were asked to complete a uniform questionnaire containing questions regarding histories of current and previous illnesses and medical treatments. The ESS questionnaire was completed before a subject underwent overnight PSG. The questionnaire and PSG data were collected and analyzed by two independent investigators. None of the subjects had undergone PSG previously. Patients who had been diagnosed or treated for OSA were excluded. Additionally, we excluded patients who had congestive heart failure, intrinsic pulmonary diseases, drug dependence, alcoholism, severe psychiatric disturbance, chronic kidney diseases, pregnancy, and those undergoing systemic steroid treatment or hormone-replacement therapy. Patients with sleep disorders other than OSA, such as upper airway resistance syndrome, restless leg syndrome, or narcolepsy, were also excluded. Subjects, whose clinical data could not be obtained, were excluded with the consistent decision of all the authors.

### Anthropometric measurements and Epworth Sleepiness Scale Questionnaire

Body habitus was measured in light clothing and bare feet by standard anthropometric methods. Waist circumference (WC) was measured midway between the lower costal margin and iliac crest, and the hip circumference (HC) was measured as the maximal girth at the greater trochanters. The NC was measured at the level of the cricothyroid membrane while the subject was standing. These data were recorded as the mean of two measurements. Body mass index (BMI) was calculated as weight divided by height squared (kg/m^2^).

The ESS is a self-administered questionnaire that provides a measure of subjective daytime sleepiness. The ESS comprises questions about subjective sleepiness in eight situations which is translated from the original edition and has been validated [18]. Respondents use a four-point scale (scored 0 to 3) to respond to each of the eight questions, and the scores are summed to give an overall score of 0 to 24.

### Polysomnography and definitions

The laboratory-based PSG is considered to be the gold standard for diagnosis of OSA. PSG (Alice 4™: Respironics Inc., Pittsburgh, USA) records were staged manually according to standard criteria by the same skilled technician. Respiratory events were scored according to the American Academic Sleep Medicine (AASM) criteria: apnea was defined as complete cessation of airflow lasting for 10 s or more; hypopnea was defined as either a ≥50% reduction in airflow for 10 s or more, or a <50% but discernible reduction in airflow accompanied either by a decrease in oxyhemoglobin saturation of ≥4% or an arousal [19]. AHI was defined as the number of events of apnea and hypopnea per hour during sleep time, based on the results of the overnight PSG. Subjects were divided into OSA group (AHI ≥5/h) and simple snoring group (AHI <5). The scorers were blinded to the ESS results.

### Statistical analysis

Continuous variables are presented as means (standard deviation, SD), except for skewed variables, which are presented as medians (interquartile range). Categorical variables are expressed as percentages. Differences between baseline characteristics of subjects in the test and validation cohorts were examined using Mann–Whitney U tests, Fisher's exact tests, Wilcoxon sum rank tests or χ^2^ tests where appropriate. Correlations between the various variables and PSG parameters were analyzed using Spearman's correlation test. The forward conditional logistic regression analysis was performed to select the OSA-related variables. The accuracy of ESS and the diagnostic model compared to PSG were examined using a receiver operating characteristics curve (ROC) to identify participants as having undiagnosed OSA. Sensitivity, specificity, positive predictive value (PPV), negative predictive value (NPV), positive and negative likelihood ratios and overall test accuracy were calculated for both the ESS and the diagnostic model to determine the cut-off values that provided maximum diagnostic efficiency.

We considered *P* values less than 0.05 to indicate statistical significance for a two-sided test. Statistical analyses were performed using the SPSS software for Windows (ver. 13.0.0; SPSS Inc., Chicago, IL, USA).

## Results

**Figure 1:**
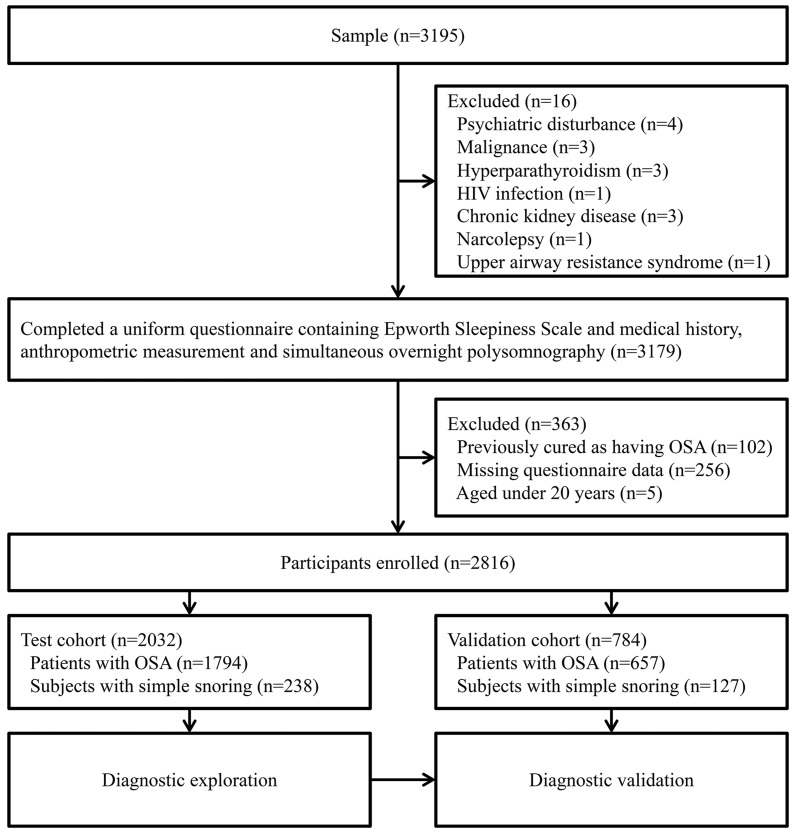
Flow diagram of recruitment of participants. HIV = Human Immunodeficiency Virus; ESS = Epworth Sleepiness Scale; OSA = obstructive sleep apnea.

A total of 3,195 consecutive Chinese subjects participated in the study. We excluded 379 individuals: 16 patients with various illnesses, 102 previously treated for OSA, 256 with missing questionnaire data, and five aged less than 20 years. We divided the remaining 2,816 participants into two cohorts: 2,032 subjects, recruited from January 2007–July 2011 were included in the test cohort, and 784 participants, recruited from August 2011–July 2012, were included in the validation cohort (Figure 1). The basic demographics and clinical characteristics of the participants are shown in Table 1 and Table S1 in File S1. We found significant differences in the values of age, BMI, NC, WC, HC, waist hip ratio, ESS score and AHI between males and females in each cohort. Females had lower NC, WC, HC, waist hip ratio, BMI, ESS score and AHI values than males (all *P*<0.01).

**Table 1:** Clinical characteristics of the participants.

	Test cohort			Validation cohort		
Clinical characteristics	Total(n = 2,032)	Male(n = 1,674)	Female(n = 358)*	Total(n = 784)	Male(n = 652)	Female(n = 132)*
Sex (%)	_	82.38	17.62	_	83.2	16.8
Age (years)	43.0 (27.0–64.0)	41.0 (27.0–63.0)	53.0 (27.0–71.0)	41.0 (27.0–64.8)	40.0 (28.0–63.0)	52.0 (24.7–68.1)
High(m)	1.70 (1.57–1.81)	1.72 (1.63–1.82)	1.60 (1.50–1.69)	1.71 (1.58–1.80)	1.72 (1.64–1.81)	1.60 (1.51–1.69)
Weight(kg)	77.0 (57.0–100.0)	80.0 (63.0–100.0)	64.0 (50.0–85.0)	77.9 (57.2–100.0)	80.0 (64.0–100.0)	64.0 (47.7–89.4)
BMI (kg/m^2^)	26.4 (21.3–33.0)	26.6 (22.0–33.2)	25.35±3.75	26.6 (21.1–32.9)	26.8 (22.0–32.8)	25.1 (18.7–33.1)
NC (cm)	40.0 (34.0–44.0)	40.0 (36.0–45.0)	36.0 (31.0–41.0)	39.0 (33.0–45.0)	40.0 (36.0–45.0)	35.0 (30.3–41.0)†
WC (cm)	95.0 (79.0–112.0)	96.0 (82.0–112.0)	88.95±10.11	95.0 (79.0–114.0)	96.3 (82.7–114.0)	88.5 (70.0–114.0)
HC (cm)	100.0 (91.0–112.0)	100.5 (92.0–112.0)	98.0 (88.0–112.0)	100.0 (90.0–113.0)	100.0 (90.0–113.0)	97.0 (84.3–112.4)
W/H ratio	0.95 (0.84–1.04)	0.95 (0.87–1.04)	0.90±0.06	0.95 (0.85–1.04)†	0.96 (0.87–1.05)†	0.91 (0.80–1.03)
ESS	9 (0–19)	10 (1–20)	7 (0–18)	9 (0–19)	10 (0–19)	7 (0–18)
SaO_2_ (%)	80.0 (55.0–95.0)	79.0 (54.0–94.0)	85.0 (60.0–96.0)	80.0 (51.0–98.0)	78.0 (50.0–98.0)	86.0 (53.7–98.0)

BMI = body mass index; NC = neck circumference; WC = waist circumference; HC = hip circumference; W/H ratio = the ratio of waist circumference to that of the hips; ESS = Epworth Sleepiness Scale; AHI = apnea-hypopnea index.

* Male *versus* female subjects in the same cohort, *P*<0.01.

† Test cohort *versus* validation cohort, *P*<0.05.

Values are presented as median (95% confidence interval) unless stated otherwise.

We examined the relationship between subjective EDS and sleep variables evaluated by PSG for all subjects in the test cohort. ESS score was significantly correlated with AHI in the OSA group (r = 0.43, *P*<0.001). However, these two variables were not significantly correlated in the simple snoring group (*P*>0.05).

**Figure 2:**
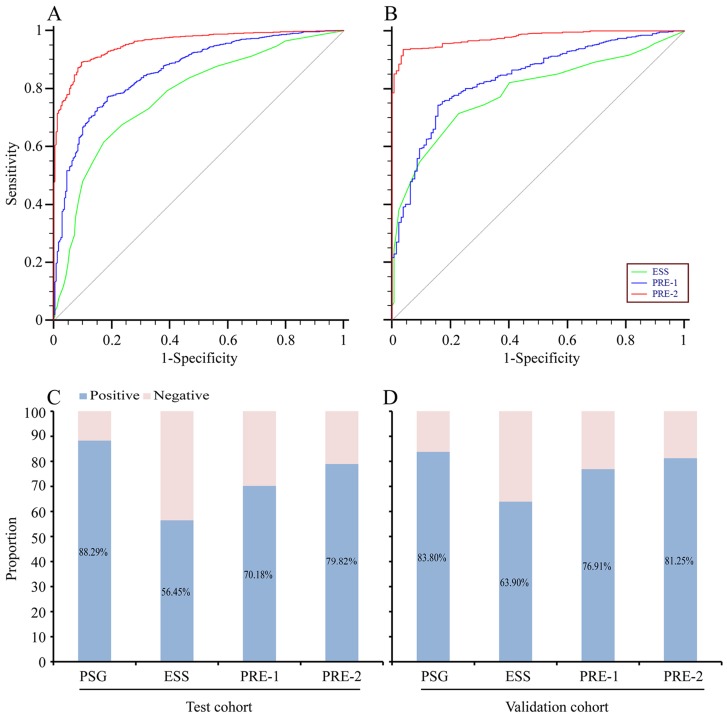
Diagnostic outcomes for Epworth Sleepiness Scale, PRE-1 and PRE-2 in screening OSA. (A) ROC curve for ESS, PRE-1 or PRE-2 *versus* PSG in the test cohort. (B) ROC curve for ESS, PRE-1 or PRE-2 *versus* PSG in the validation cohort. (C) Rate of positive results for PSG, ESS, PRE-1 or PRE-2 in the test cohort. (D) Rate of positive results for PSG, ESS, PRE-1 or PRE-2 in the validation cohort. OSA = obstructive sleep apnea; ESS = Epworth Sleepiness Scale; PRE-1 = the predictive variable for the first diagnostic model; PRE-2 = the predictive variable for the second diagnostic model; ROC curve = the receiver operating characteristics curve; PSG = polysomnography.

**Figure 3:**
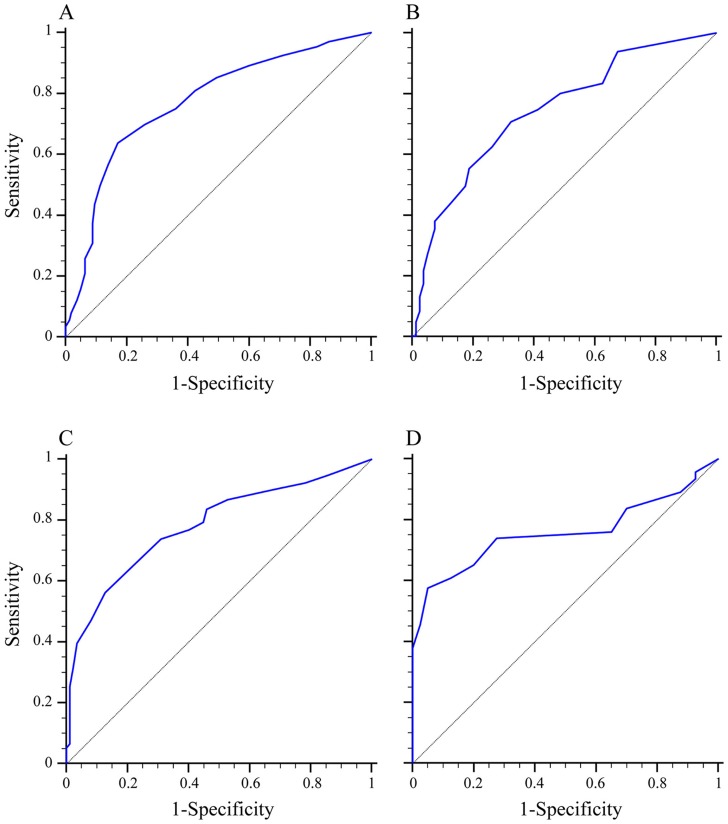
Diagnostic outcomes for ESS on different sexes in the diagnosis of OSA. (A) ROC curve for ESS *versus* PSG for male subjects in the test cohort (AUC = 0.771). (B) ROC curve for ESS *versus* PSG for female subjects in the test cohort (AUC = 0.744). (C) ROC curve for ESS *versus* PSG for male subjects in the validation cohort (AUC = 0.781). (D) ROC curve for ESS *versus* PSG for female subjects in the validation cohort (AUC = 0.759). ESS = Epworth Sleepiness Scale; OSA = obstructive sleep apnea; ROC curve = the receiver operating characteristics curve; PSG = polysomnography; AUC = area under the ROC curve.

ROC curves showed that the optimum diagnostic cut-off point of ESS for the total subjects was nine, the area under the ROC curve (AUC) was 0.774 (95% confidence interval [CI], 0.743–0.805, sensitivity 61.65%, specificity 82.77%; Table 2, Figure 2A). Remarkably, we found the optimum diagnostic cut-off point for ESS differed with respect to sex: nine for males *versus* six for females. The corresponding AUCs were 0.771 (95% CI, 0.750–0.791) for males and 0.744 (95% CI, 0.695–0.788) for females (Table 2, Table S2 in File S1 and Figure 3). There was no significant difference between these two AUCs (*P*>0.05). However, we obtained a moderate sensitivity in the test cohort of 61.65% (95% CI, 59.4–63.9) and a specificity of 82.77% (95% CI, 77.4–87.3) for detection of undiagnosed OSA when the ESS threshold number was set at nine. To validate these data, we divided the subjects into OSA and non-OSA groups in the validation cohort using the optimum diagnostic cut-off points of ESS score: nine for the whole group, nine for males, and six for females. Results in the validation cohort were similar to those in the test cohort (Table S3 and Table S4 in File S1).

**Table 2:** The efficiency for detecting OSA with ESS *versus* PSG for the total subjects (n = 2,032), male subjects (n = 1,674) and female subjects (n = 358) in the test cohort.

Criterion	Best cut-off point	AUC	Sensitivity (%)	Specificity (%)	+LR	−LR	+PV (%)	−PV (%)
Total	9	0.774 (0.743–0.805)	61.65 (59.35–63.91)	82.77 (77.36–87.35)	3.58 (2.70–4.74)	0.46 (0.43–0.50)	96.4 (95.18–97.42)	22.3 (19.56–25.15)
Male	9	0.771 (0.750–0.791)	63.85 (61.38–66.27)	82.91 (76.12–88.43)	3.74 (2.64–5.28)	0.44 (0.40–0.48)	97.29 (96.08–98.20)	19.29 (16.39–22.47)
Female	6	0.744 (0.695–0.788)	70.86 (65.14–76.14)	67.50 (56.11–77.55)	2.18 (1.58–3.02)	0.43 (0.34–0.55)	88.34 (83.38–92.24)	40.00 (31.67–48.78)

PSG = polysomnography; ESS = Epworth Sleepiness Scale; AUC = area under the ROC curve; +LR = Positive likelihood ratio; −LR = Negative likelihood ratio; +PV = Positive predictive value; −PV = Negative predictive value.

Values in parentheses are 95% confidence intervals.

Because the diagnostic accuracies of ESS alone in both the test and validation cohorts were only fair, we developed a new screening model by combining other parameters to improve the OSA diagnostic efficiency. First, we divided all participants in the test cohort into OSA and non-OSA groups, according to the PSG record. Then, we included all the significant variables listed in Table 1 to identify the parameters independently associated with an increased likelihood of OSA. Initially, minimum SaO_2_ was not included in the model due to the need for professional equipment. Through the forward conditional logistic regression analysis, five clinical variables (age, BMI, WC, ESS and sex) were evaluated as independent predictors of OSA. Next, we generated a model: P_1_ = 1/[1+ exp (0.048× age +0.135× BMI +0.061× WC +0.162× ESS +0.454× sex –10.535)]. The value of sex was set as 0 for female and 1 for male. The probability of OSA, named PRE-1, for a given patient can be obtained by applying the following formula, 1/(1+ e ^–sum^), which displays the relationship between the sum and predicted probability of OSA. Sum is the linear combination of model coefficients multiplied by the values of the respective variables. Consequently, the AUC was 0.861 (95% CI, 0.845–0.876) for diagnosis of OSA with the new predictive variable, PRE-1. This showed a sensitivity of 77.03% (95% CI, 75.0–79.0) and a specificity of 81.51% (95% CI, 76.0–86.2) to the optimum diagnostic cut-off point for PRE-1 (PRE-1 = 0.8822) (Table 3 and Table S2 in File S1).

**Table 3:** The efficiency for detecting OSA with PRE-1 and PRE-2 *versus* PSG in the test cohort (n = 2,032).

Criterion	AUC*	Sensitivity (%)	Specificity (%)	+LR	−LR	+PV (%)	−PV (%)
PRE-1	0.861 (0.845–0.876)	77.03 (75.02–78.96)	81.51 (75.99–86.23)	4.17 (3.19–5.45)	0.28 (0.25–0.31)	96.91 (95.88–97.75)	32.01 (28.31–35.89)
PRE-2	0.955 (0.946–0.964)	89.13 (87.60–90.53)	90.34 (85.85–93.77)	9.22 (6.25–13.61)	0.12 (0.10–0.14)	98.58 (97.88–99.10)	52.44 (47.48–57.36)

PRE-1 = the predictive variable for the first diagnostic model; PRE-2 = the predictive variable for the second diagnostic model; PSG = polysomnography; AUC = area under the ROC curve; +LR = Positive likelihood ratio; −LR = Negative likelihood ratio; +PV = Positive predictive value; −PV = Negative predictive value.

* The AUCs of ESS, PRE-1 and PRE-2 compared in pairs, *P*<0.0001.

Values in parentheses are 95% confidence intervals.

With the prevalence of portable pulse oximetry tools, minimum SaO_2_ can be easily assessed in most clinical practices. Based on the previous analysis, we determined whether minimum SaO_2_ should be included in the multiple logistic regression model using the forward conditional logistic regression method. Then, we generated a model that included four variables (age, WC, ESS and minimum SaO_2_) as independent predictors of OSA. Using the same procedure, we generated another model: P_2_ = 1/[1+ exp (0.029× age +0.059× WC +0.111× ESS –0.359× minimum SaO_2_ +26.202)]. The probability of OSA was named PRE-2. The AUC was 0.955 (95% CI, 0.946–0.964) for OSA diagnosis using PRE-2. It showed a sensitivity of 89.13% (95% CI, 87.6–90.5) and a specificity of 90.34% (95% CI, 85.9–93.8) to the optimum diagnostic cut-off point for PRE-2 (PRE-2 = 0.8294) (Table 3, Figure 2A and Table S2 in File S1).

With the pairwise comparison analysis, the areas under the ROC curves at the best cut-off point for ESS alone, PRE-1 and PRE-2 were significantly different (*P*<0.0001). So we selected PRE-2 as the final screening model, due to its greater accuracy in the test cohort. Then, we entered the data of the validation cohort into the final model, and calculated the corresponding PRE-2 value. First, we validated the accuracy of the final screening model with the calculated PRE-2 of the validation cohort. The corresponding AUC was 0.977 (95% CI, 0.964–0.986) for the final model (Table 4). Second, we assessed the accuracy of the optimum diagnostic cut-off point (PRE-2 = 0.8294) obtained from the test cohort. We divided the subjects in the validation cohort into OSA and non-OSA groups using the optimum diagnostic cut-off point (PRE-2 = 0.8294), and compared the outcomes with the PSG records (Table S4 in File S1); sensitivity was 94.22% (95% CI, 92.15–95.87) and specificity was 85.83% (95% CI, 78.53–91.38) (Table 4). The outcomes of PRE-1 in the validation cohort were also calculated, as shown in Figure 2B and Table 4.

**Table 4:** The efficiency for detecting OSA with ESS, PRE-1 and PRE-2 *versus* PSG in the validation cohort (n = 784).

Criterion	AUC*	Sensitivity (%)	Specificity (%)	+LR	−LR	+PV (%)	−PV (%)
ESS	0.790 (0.760–0.818)	71.54 (67.92–74.96)	75.59 (67.18–82.77)	2.93 (2.15–4.00)	0.38 (0.32–0.44)	93.81 (91.33–95.76)	33.92 (28.42–39.76)
PRE-1	0.839 (0.811–0.864)	86.91 (84.09–89.39)	74.80 (66.33–82.08)	3.45 (2.55–4.66)	0.17 (0.14–0.22)	94.69 (92.59–96.34)	52.49 (44.95–59.94)
PRE-2	0.977 (0.964–0.986)	94.22 (92.15–95.87)	85.83 (78.53–91.38)	6.65 (4.33–10.20)	0.07 (0.05–0.09)	97.17 (95.57–98.32)	74.15 (66.29–81.01)

OSA = obstructive sleep apnea; ESS = Epworth Sleepiness Scale; PRE-1 = the predictive variable for the first diagnostic model; PRE-2 = the predictive variable for the second diagnostic model; AUC = area under the ROC curve; +LR = Positive likelihood ratio; −LR = Negative likelihood ratio; +PV = Positive predictive value; −PV = Negative predictive value.

* The AUCs of ESS, PRE-1 and PRE-2 compared in pairs, *P*<0.0001.

Values in parentheses are 95% confidence intervals.

## Discussion

In the retrospective study of 2,816 subjects with suspected OSA, we demonstrated that ESS had fair specificity for screening and detecting patients with undiagnosed OSA with different ESS thresholds between males and females; we further developed a simplified screening model consisting of age, WC, ESS and minimum SaO_2_, which exhibited satisfactory efficiency in terms of identifying patients with OSA.

Historically, the ESS was designed as a method of measuring daytime sleepiness and the validity and reliability of the ESS was unaffected by cultural or language factors [20], [21]. Since EDS is a significant manifestation and an important marker for assessment of OSA, the ESS currently plays an important role in the screening process for determination of whether a patient should be referred to a sleep laboratory for PSG [22]. Many specialists have studied the efficiency of ESS compared with PSG for diagnosis of OSA [23], [24], revealing a high specificity and a low sensitivity for identification of OSA, which was replicated in our study.

Further analysis indicated that the sex composition was severely skewed (Table 1). Hence we further evaluated the cohorts using sex-stratification. Interestingly, when we divided the subjects into two groups by sex, we found the cut-off points were different between these two groups, with nine for males and six for females. This condition, with the majority of the subjects being male, has been reported previously (Table S5 in File S1). However, these studies did not perform a sex-stratified analysis or report a difference in cut-off points of ESS between the sexes. Sex differences between male and female patients with OSA have been known for a long time, and differences in terms of admission and some comorbid conditions have been reported [25], [26]. However, to our knowledge, this is the first study to investigate sex differences with respect to the ESS score threshold in OSA. In China, female patients may not always show the same clinical symptoms as male patients. Moreover, sociocultural factors, such as education status and socio-economic position, have an important impact in females. Both these reasons may result in fewer acknowledgements of EDS by females, which leads to a lower baseline self-reported EDS in females compared to that in males.

Our preliminary analysis suggested that ESS alone, reflecting the single symptom of EDS, may be insufficient to screen for OSA. To increase the diagnostic efficiency, we explored the feasibility of establishing a new model using OSA-related parameters. First, we included easily obtainable anthropometric parameters into the multiple logistic regression model, and generated a screening model that incorporated variables of age, BMI, WC, ESS and sex. The AUC for the dependent variable PRE-1 indicated a higher efficiency than the ESS alone (P<0.0001). Then, we added minimum SaO_2_ to the screening model. As one of the important indicators reflect the severity of OSA, minimum SaO_2_ is easily measured by pulse oximetry, which is commonly used in primary hospitals and family care facilities. The AUC for the dependent variable PRE-2 indicated a much higher efficiency than the former screening model (P<0.0001). These outcomes suggested the feasibility of minimum SaO_2_ in predicting OSA, though it may not present the entire pattern of oxygen saturation during sleep.

Many studies [12], [26], [27] have recommended different clinical variables to screen OSA. For example, in a prospective study evaluating AHI prediction models, Dixon *et al.* [28] reported that sensitivity was high (85–98%), but specificity was low (33–39%). Chai-Coetzer *et al.* [13] developed a model using a screening questionnaire followed by oxygen desaturation index, which had a sensitivity and specificity both above 80%. However, that study was limited because it included only patients with moderate-to-severe OSA. Additionally, the proportions of male and female patients were almost identical, which is not consistent with the sex difference in the prevalence of OSA in the general population [29]. Our study assessed a wide range of OSA severity, the sensitivity and specificity of our model were above 85% at the best classification of the predictive probability, in both the test and validation cohorts. Furthermore, the sex composition of the patients with OSA in our study was more similar to that in the general population. Notably, in recent years there is a dramatic shift from PSG to home sleep testing for OSA which might reduce the price and effort of OSA diagnosis [30]. However, in China, a developing country with the largest population in the world, there is still a long way to go. Hence, our findings may indicate an auxiliary tool to reduce healthcare costs associated with OSA by facilitating diagnosis and enabling more timely therapeutic intervention.

Although our findings are based upon a relatively large sample, some limitations should be stated. Firstly, the sex composition of the subjects was severely skewed, the prevalence of OSA in participants referred to the sleep center was higher in our study cohorts than in the general population, which may limit the generalization of our findings to the general population. To eliminate the bias, we performed a sex-stratified analysis. Additionally, the sensitivity and specificity are measures of intrinsic diagnostic accuracy because they are not affected by the prevalence of the condition [31]. That is to say, although this study chose a sample of participants from sleep clinics where referral from prevalence of OSA is high, the consequences of ROC curve are credible. Secondly, ESS is a self-administered questionnaire, and may not provide good reliability due to its subjective nature [32]. We excluded those subjects with psychological or psychiatric illness. All participants completed the ESS questionnaires before the night-time PSG test, which might reduce the bias in the outcome. Thirdly, other factors, such as ethnicity [33], or menopausal status in females, may affect sleepiness severity in patients with suspected OSA. Considering these limitations, further study is warranted to confirm the diagnostic efficiency of the models in different countries and ethnic groups.

## Conclusions

In conclusion, we found that the ESS was moderately useful for screening undiagnosed OSA in adult population, and there may exist sex differences in ESS score cut-offs. The high sensitivity and specificity of the final screening model indicated a simple and practical method for screening adults at high risk of OSA. This will facilitate targeted initiation of preventive measures for minimizing the major health-related consequences in China.

## Supporting Information

File S1 **File containing Tables S1–S5.** Table S1. The formation of the participants in the test cohort and validation cohort based on the severity of OSA and sexes. Table S2. Results of different models in diagnosing OSA comparing with PSG in the test cohort (n = 2,032). Table S3. The efficiency for detecting OSA with ESS *versus* PSG for the male subjects (n = 652) and female subjects (n = 132) in the validation cohort. Values in parentheses are 95% confidence intervals. Table S4. Results of different models in diagnosing OSA comparing with PSG in the validation cohort (n = 784). Table S5. Information for the former studies related to the cut-off point of Epworth Sleepiness Scale.
(DOC)
