# Supplementary material for: An Effective Model for Screening Obstructive Sleep Apnea: A Large-Scale Diagnostic Study
Source: PLoS One. 2013 Dec 2;8(12):e80704. doi: 10.1371/journal.pone.0080704 (PMC3846620; doi:10.1371/journal.pone.0080704)
Supplement: File S1 — File containing Tables S1–S5. Table S1. The formation of the participants in the test cohort and validation cohort based on the severity of OSA and sexes. Table S2. Results of different models in diagnosing OSA comparing with PSG in the test cohort (n = 2,032). Table S3. The efficiency for detecting OSA with ESS versus PSG for the male subjects (n = 652) and female subjects (n = 132) in the validation cohort. Values in parentheses are 95% confidence intervals. Table S4. Results of different models in diagnosing OSA comparing with PSG in the validation cohort (n = 784). Table S5. Information for the former studies related to the cut-off point of Epworth Sleepiness Scale. (DOC) [file pone.0080704.s001.doc]

## Supplementary file S1

## An effective model for screening obstructive sleep apnea: A large-scale diagnostic study

Jianyin Zou, Jian Guan, Hongliang Yi, Lili Meng, Yuanping Xiong, Xulan Tang, Kaiming Su, and Shankai Yin

**Supplementary Tables**

**Table S1. The formation of the participants in the test cohort and validation cohort based on the severity of OSA and sexes**

|  | Test cohort(n=2,032) | | Validation cohort(n=784) | |
| --- | --- | --- | --- | --- |
| All of the participants (n=2,816) | Male(n=1,674) | Female(n=358) | Male(n=652) | Female(n=132) |
| Simple snoring (n=365) | 158 | 80 | 87 | 40 |
| Mild OSA (n=470) | 255 | 87 | 98 | 30 |
| Moderate OSA (n=471) | 268 | 72 | 111 | 20 |
| Severe OSA (n=1510) | 993 | 119 | 356 | 42 |

Simple snoring was defined as AHI<5; mild OSA was defined as AHI between 5 and 15 per hour; moderate OSA was defined as AHI between 15 and 30 per hour; severe OSA was defined as AHI>30 per hour.

OSA=obstructive sleep apnea.

Table S2. Results of different models in diagnosing OSA comparing with PSG in the test cohort (n=2,032)

|  | PSG | |  |
| --- | --- | --- | --- |
|  | Positive | Negative | Total |
| ESS |  | | |
| Positive | 1106 | 41 | 1147 |
| Negative | 688 | 197 | 885 |
| Total | 1794 | 238 | 2032 |
| PRE-1 |  | | |
| Positive | 1382 | 44 | 1426 |
| Negative | 412 | 194 | 606 |
| Total | 1794 | 238 | 2032 |
| PRE-2 |  | | |
| Positive | 1599 | 23 | 1622 |
| Negative | 195 | 215 | 410 |
| Total | 1794 | 238 | 2032 |

OSA=obstructive sleep apnea; PSG=polysomnography; ESS=Epworth Sleepiness Scale; PRE-1=the predictive variable for the first diagnostic model; PRE-2=the predictive variable for the second diagnostic model.

Table S3. The efficiency for detecting OSA with ESS *versus* PSG for the male subjects (n=652) and female subjects (n=132) in the validation cohort. Values in parentheses are 95% confidence intervals

| Criterion | threshold | AUC* | Sensitivity (%) | Specificity (%) | +LR | -LR | +PV (%) | -PV (%) |
| --- | --- | --- | --- | --- | --- | --- | --- | --- |
| ESS | 9 | 0.790 (0.760-0.818) | 71.54 (67.92-74.96) | 75.59 (67.18-82.77) | 2.93 (2.15-4.00) | 0.38 (0.32-0.44) | 93.81 (91.33-95.76) | 33.92 (28.42-39.76) |
| Male | 9 | 0.781 (0.747-0.812) | 73.81 (69.97-77.39) | 68.97 (58.14-78.45) | 2.38 (1.73-3.27) | 0.38 (0.31-0.46) | 93.92 (91.27-95.95) | 28.85 (22.79-35.52) |
| Female | 6 | 0.759 (0.677-0.829) | 73.91 (63.71-82.51) | 70.00 (53.47-83.42) | 2.46 (1.51-4.02) | 0.37 (0.25-0.56) | 85.00 (75.26-91.99) | 53.85 (29.47-67.76) |

*AUC was analyzed again to estimate the accuracy of ESS for diagnosing OSA in the validation cohort.

ESS=Epworth Sleepiness Scale; AUC=area under the ROC curve; +LR=Positive likelihood ratio; -LR=Negative likelihood ratio; +PV=Positive predictive value; -PV=Negative predictive value.

Table S4. Results of different models in diagnosing OSA comparing with PSG in the validation cohort (n=784)

|  | PSG | |  |
| --- | --- | --- | --- |
|  | Positive | Negative | Total |
| ESS |  | | |
| Positive | 470 | 31 | 501 |
| Negative | 187 | 96 | 283 |
| Total | 657 | 127 | 784 |
| PRE-1 |  | | |
| Positive | 571 | 32 | 603 |
| Negative | 86 | 95 | 181 |
| Total | 657 | 127 | 784 |
| PRE-2 |  | | |
| Positive | 619 | 18 | 637 |
| Negative | 38 | 109 | 147 |
| Total | 657 | 127 | 784 |

OSA=obstructive sleep apnea; PSG=polysomnography; ESS=Epworth Sleepiness Scale; PRE-1=the predictive variable for the first diagnostic model; PRE-2=the predictive variable for the second diagnostic model.

Table S5. Information for the former studies related to the cut-off point of Epworth Sleepiness Scale

| Study | Journal | Sample (male/female) | Method | Cut-off ponit |
| --- | --- | --- | --- | --- |
| Johns,1993 | Chest | 273 (247/26) | Observation | 10 |
| Rosenthal, 2008 | J NERV MENT DIS | 268 (180/88) | ROC curve | 8 |
| Chan, 2009 | Sleep Breath | 192 (136/56) | ROC curve | 8 |
| Kopitovic, 2010 | Sleep Breath | 223 (112/111)* | ROC curve | 9 |

* Only 25(22%) female patients in case group.

ROC curve=the receiver operating characteristics curve.
